# Can video playback provide social information for foraging blue tits?

**DOI:** 10.7717/peerj.3062

**Published:** 2017-03-21

**Authors:** Liisa Hämäläinen, Hannah M. Rowland, Johanna Mappes, Rose Thorogood

**Affiliations:** 1 Department of Zoology, University of Cambridge, Cambridge, United Kingdom; 2 Institute of Zoology, Zoological Society of London, London, United Kindgom; 3 Centre of Excellence in Biological Interactions, Department of Biological and Environmental Science, University of Jyväskylä, Jyväskylä, Finland; 4 Department of Biosciences, University of Helsinki, Helsinki, Finland

**Keywords:** Blue tits, Social information use, Video-playback

## Abstract

Video playback is becoming a common method for manipulating social stimuli in experiments. Parid tits are one of the most commonly studied groups of wild birds. However, it is not yet clear if tits respond to video playback or how their behavioural responses should be measured. Behaviours may also differ depending on what they observe demonstrators encountering. Here we present blue tits (*Cyanistes caeruleus*) videos of demonstrators discovering palatable or aversive prey (injected with bitter-tasting Bitrex) from coloured feeding cups. First we quantify variation in demonstrators’ responses to the prey items: aversive prey provoked high rates of beak wiping and head shaking. We then show that focal blue tits respond differently to the presence of a demonstrator on a video screen, depending on whether demonstrators discover palatable or aversive prey. Focal birds faced the video screen more during aversive prey presentations, and made more head turns. Regardless of prey type, focal birds also hopped more frequently during the presence of a demonstrator (compared to a control video of a different coloured feeding cup in an empty cage). Finally, we tested if demonstrators’ behaviour affected focal birds’ food preferences by giving individuals a choice to forage from the same cup as a demonstrator, or from the cup in the control video. We found that only half of the individuals made their choice in accordance to social information in the videos, i.e., their foraging choices were not different from random. Individuals that chose in accordance with a demonstrator, however, made their choice faster than individuals that chose an alternative cup. Together, our results suggest that video playback can provide social cues to blue tits, but individuals vary greatly in how they use this information in their foraging decisions.

## Introduction

Potential prey items differ in their nutritional benefits and palatability (Skelhorn, Halpin & Rowe, 2016). Therefore, when foraging, animals face decisions that require an estimation of the profitability of prey (Pyke, Pulliam & Charnov, 1977), in order to maximize their fitness. As well as learning from their own experience with prey (Skelhorn, Halpin & Rowe, 2016), predators can gather social information from observing the foraging experiences of others (Galef & Giraldeau, 2001). For example, observing conspecifics consuming palatable food positively influences food preferences in many avian species (Mason & Reidinger, 1981; McQuoid & Galef, 1993; Fryday & Greig-Smith, 1994). However, the potential for information to be available from observing an encounter with less palatable prey has received far less attention.

Many bird species show a clear disgust response by vigorously wiping their beaks on a perch (Clark, 1970; Ganchrow, Steiner & Bartana, 1990). This cue might provide social information about the profitability of food resources to others. For example, young chicks that observe beak wiping and head shaking are less likely to peck at, or consume, the same foods (Johnston, Burne & Rose, 1998; Skelhorn, 2011), and red-winged blackbirds will avoid feeding cups if demonstrators are induced to vomit after eating (Mason & Reidinger, 1982). Parid tits are one of the most studied wild birds in Europe, with an increasing focus on their social behavior and learning (e.g., Sasvári, 1979; Marchetti & Drent, 2000; Aplin, Sheldon & Morand-Ferron, 2013). While previous studies have shown that parid tits can learn a novel foraging task by observing other individuals (Sasvári, 1979; Aplin, Sheldon & Morand-Ferron, 2013), it is not yet known how they use social information about food palatability in their foraging decisions.

Our first aim was to investigate how wild-caught blue tits (*Cyanistes caeruleus*) use information from foraging conspecifics, and if their response differs depending on the palatability of food that demonstrators encounter. However, when studies involve experimentally manipulating social behaviour, it can be difficult to control what stimuli focal birds observe (McQuoid & Galef, 1993). Issues can also arise because of social characteristics of the birds themselves (e.g., dominance, Nicol & Pope, 1999). Video playback might circumvent these issues and provide many advantages over live demonstrators. With videos, it is possible to manipulate the characteristics presented and control the timing of the video presentation, thus enabling controlled and standardized stimuli to be presented to focal individuals (D’Eath, 1998; Woo & Rieucau, 2011). Furthermore, use of video playback also has ethical implications. For instance, using video playback to study how individuals use social information about food palatability requires fewer demonstrators to be encouraged to eat unpalatable food (e.g., Mason & Reidinger, 1982).

Video playback also has potential shortcomings, however, that should be considered when using these stimuli in behavioural studies (D’Eath, 1998; Woo & Rieucau, 2011). For instance, in many species, physical interactions between an observer and a demonstrator play an important role in certain behaviours, such as in aggressive contests and courtship, so the applicability of video playback in these contexts may be limited (D’Eath, 1998). To be able to study focal individuals’ responses to specific stimuli, we also need to be sure that individuals pay attention to subtleties in demonstrators’ behaviour instead of simply responding to their presence in a video (McQuoid & Galef, 1993). To date, video presentations have been used successfully in both captive (Rowland et al., 1995; Ord et al., 2002; Bird & Emery, 2008) and field studies (Clark, Macedonia & Rosenthal, 1997; Burford, McGregor & Oliveira, 2000; Gunhold, Whiten & Bugnyar, 2014) across different contexts, including studies of mate preference (e.g., Ophir & Galef, 2003), social learning (e.g., McQuoid & Galef, 1993), and predator recognition (e.g., Evans, Macedonia & Marler, 1993), and for a range of taxa, including mammals (Gunhold, Whiten & Bugnyar, 2014), fish (Rowland et al., 1995), lizards (Ord et al., 2002), spiders (Clark & Uetz, 1992), and birds (Rieucau & Giraldeau, 2009; Zoratto et al., 2014). Surprisingly, however, the validity of video playback has not been tested for parid tits. Therefore, the second aim of our study was to investigate its applicability.

In this study, we presented focal birds with videos of a demonstrator encountering palatable and aversive prey items in novel, coloured feeding cups. We first quantified variation in demonstrator blue tits’ behaviour when encountering these two prey types before presenting standardized videos of these encounters to focal birds. These videos also included a control section that consisted of a different coloured feeding cup in an empty cage; we predicted that individuals would pay more attention to the videos when a conspecific was present. The control section was presented to focal birds both before and after a demonstrator appeared on the screen to investigate if birds’ response to control cups would change after they had seen a demonstrator foraging from a different cup. Because demonstrators were more active during the encounter with aversive prey, we also predicted that this might provide more cues and therefore elicit more vigorous response in focal birds, compared to a video of palatable prey.

Recent studies have shown that acquiring information by observing others does not always result in use of that social information (Carter et al., 2014; Mesoudi et al., 2016). To investigate whether video playback can be used to manipulate social information for blue tits, we used a simple choice test to record if observers preferred to feed from a similarly coloured cup as the demonstrator, or from the different coloured cup (present in the control video). We predicted that focal birds observing a demonstrator encountering a palatable prey, would choose to feed from the same cup as the demonstrator, whereas observation of an encounter with aversive prey would lead them to avoid the cup in which the demonstrator found the distasteful prey item.

Finally, familiarity with the demonstrator may influence responses to playback. Previous studies have shown that Japanese quail (*Coturnix japonica*) females identify specific males that they see in videos (Ophir & Galef, 2003) and that rooks (*Corvus frugilegus*) spend more time looking at a video of their partner, compared to a video of a nonaffiliated conspecific (Bird & Emery, 2008). The value of social information may also vary depending on familiarity (Firth, Sheldon & Farine, 2016; Mesoudi et al., 2016) or previous experiences (Farine, Spencer & Boogert, 2015), with the demonstrator. We attempted to account for this by including a measure of association strength from our study population’s social network, and predicted that the identity of the demonstrator would influence the behavioural responses of observers.

## Methods

### Birds and housing

The study was conducted from January to March 2016 at Madingley Wood (0°3.2′E, 52°12.9′N), an established study site in Cambridge, UK. There is an ongoing long-term study of great tit (*Parus major*) and blue tit populations in the area and birds have been given British Trust of Ornithology (BTO) ID rings and fitted with small passive integrated transponder (PIT) tags (fitted to a colour ring) since 2012. In January, five sunflower seed feeders were fitted with PIT tag reading antennae and data loggers that scanned birds’ unique PIT tag codes when they landed on a feeder. During the winter, great tits and blue tits form loose fission–fusion flocks that move between food sources (Ekman, 1989). This flocking behaviour allows us to use the records from the feeders to identify individuals that forage in the same flock. We used a Gaussian mixture model to detect these gathering events (Psorakis et al., 2012), and then calculated social associations (i.e., edge weights in the social network) between individuals based on how often they were present in the same group (gambit of the group approach, Franks, Ruxton & James, 2010).

Wild blue tits (*n* = 25) were captured with mist nets (by HMR) in February 2016. Individuals were chosen from the population randomly, but PIT tag records enabled us to calculate association strengths for each observer-demonstrator pair used in the experiment. All captured birds were adults (based on plumage), but their sex was unknown. Birds were housed indoors in individual plywood cages (80 cm × 65 cm × 50 cm) with a daily light period of 12 h. Food (sunflower seeds, peanuts and tallow) and water were provided ad libitum except prior and during the experiment when food restriction was necessary. This restriction lasted no longer than one hour, following guidelines to the operation of the Animal (Scientific Procedures) Act 1986 (2009), and the Association for the Study of Animal Behaviour’s Guidelines for the treatment of animals in behavioural research and teaching (2012). Birds were kept in captivity for a maximum of four days until released at the capture site, and they were in auditory (but not visual) contact during housing and experiments. The study was conducted under existing Home Office (PPL 60/4322) and Natural England (2015-6665-SCI-SCI-3) licenses held by HMR.

### Experimental protocol

#### Prey types

We created two types of prey: a palatable mealworm and an aversive mealworm that was injected and coated with 2.5% solution of denatonium benzoate (Bitrex). Bitrex tastes bitter to humans (Chandrashekar et al., 2000) and elicits beak wiping in birds (Skelhorn & Rowe, 2009).

#### Video recording

We used six individual blue tits as demonstrators for the videos. Three of these birds were first used as observers in the social information use test before recording them as demonstrators. Birds were moved from their home cage to wooden test cages (66 cm × 50 cm × 50 cm) that differed from the home cage in that they had a front wall made of plexiglass. We coated the plexiglass with tinted film that made it possible to observe and film the birds while minimising effects on their behaviour

We first filmed the demonstrators eating a palatable mealworm in a coloured feeding cup (yellow or green). We then filmed the same individual eating a Bitrex mealworm in a different coloured cup (blue or purple). Because an experience of prey with chemical defences was likely to affect birds’ response towards palatable prey and their willingness to consume it, we always filmed responses towards palatable prey first.

From the videos, we quantified differences in demonstrators’ response to palatable and aversive prey from first contact with the prey item until 10 s after eating. We measured (i) how long demonstrators spent wiping their beaks on a perch (in seconds), (ii) the number of beak wipes they performed, and (iii) the number of times the head was shaken. We then used these videos to create standardized videos to present to observers (see Videos S1 and S2).

#### Video presentation

All videos included 45 s of a demonstrator finding a prey item in a coloured cup and a demonstrator’s response to that prey. In addition, each video included 60 s of a different coloured cup in an empty cage to make sure that observers were familiar with both cup colours and their foraging choice would depend on the information in the video instead of novelty of cups. Thirty seconds of this control video was shown to observers before a demonstrator appeared on the screen and 30 s was shown after a demonstrator, as we predicted that observers might pay attention to the cups differently after seeing a foraging conspecific. We used green and yellow cups when demonstrators encountered palatable prey (randomizing which cup colour was shown with a demonstrator and which in an empty cage), and blue and purple cups when demonstrators encountered aversive prey. We showed each observer (*n* = 22) two videos, once for each prey type. These two videos were shown on sequential days and the order was randomized among birds. We did not change the demonstrator between different prey types, so observers saw the same demonstrator encounter both palatable and aversive prey. Therefore, we think that any differences in responses of the observers are likely due to the prey type, not the demonstrator’s identity.

**Figure 1:**
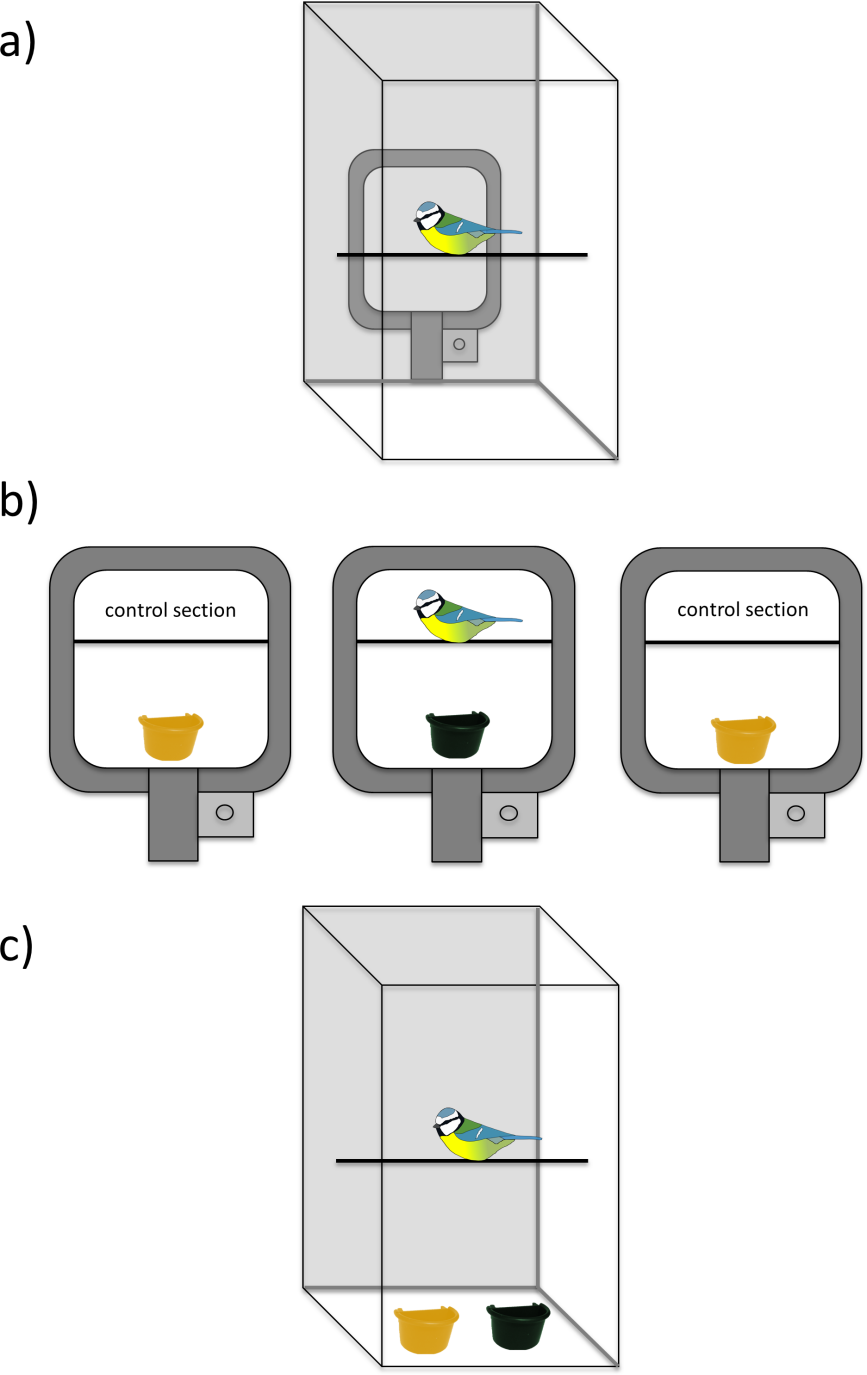
The experimental set-up. (A) The view of the test cage (a computer monitor and a camera recording focal birds’ behaviours were placed against a plexiglass wall of the cage), (B) presentation of video playback including a demonstrator discovering prey from a coloured feeding cup (45 s) and two control sections of a different coloured cup (30 s before and 30 s after a demonstrator), (C) social information use test, where focal birds had a choice to forage from the same cup as the demonstrator, or from the cup in the control video.

Observers were moved to a test cage 2 h before the video presentations to allow habituation to the cage. We then placed a computer monitor (Dell 1908FPc, 19″) against the plexiglass front wall of the cage for 15 min before showing the video (Fig. 1). This did not seem to stress the birds and they got used to the monitor quickly. We recorded observers during the video-playback (using a DBPOWER 1080P action camera; see Video S3), so that we could classify their behaviour during the different sections of the video playback: when they were shown (i) cups before the demonstrator appeared (30 s), (ii) the demonstrator’s response to the prey (45 s), and (iii) the same cups once the demonstrator was no longer present (30 s). From these recordings, we analyzed (i) the time that a bird spent facing the screen or flying, (ii) the number of head turns indicating increased vigilance, and (iii) the number of hops a bird performed on a perch, suggested to indicate increased nervousness such as neophobia (e.g., Heinrich, 1988).

The entire cage was not in the field of view of the camera, so sometimes birds flying close to the roof of the cage, or holding on near the roof, could not be seen in the video. Therefore, we excluded individuals from our dataset that were visible for less than 30 s during the whole 105 s video (60 s of cups only and 45 s of a demonstrator). After removing these individuals, our dataset included 13 individuals that were shown videos of a demonstrator encountering both palatable and aversive prey, and 3 individuals that were visible only when they were shown a video of a demonstrator encountering palatable prey. The final sample size for video analysis was therefore 16 observers when prey was palatable (mean time that observers were visible = 81 s, range = 38–105 s) and 13 observers when it was aversive (mean time that observers were visible = 90 s, range = 43–105 s).

#### Observer social information use test

Immediately following each session of video playback, we tested if focal birds (*n* = 22) used social information from the videos by giving them a choice between two different coloured cups: the cup that a demonstrator fed from, and the cup that was shown in an empty cage without a demonstrator (Fig. 1C). Both cups were filled with sand, so that birds could not see their contents and the time cost to search for prey was higher. Before the test, focal birds had been trained in their home cages to search for food hidden in the sand by offering them mealworms in a white feeding cup. Training was done stepwise, by first offering mealworms that were clearly visible, and then covering them partly with sand, until birds learned to search for worms that were completely hidden. Focal birds were therefore familiar with the foraging task in the experiment. We filmed the focal birds during the test and from these, recorded the time that it took birds to land on a first cup, and their choice. The test was finished after birds had landed on both cups, or after 20 min. Two birds in the palatable prey test and one individual in the aversive prey test did not land on either cup in 20 min and were excluded from analyses.

### Statistical analyses

All analyses were conducted using software R 3.2.2 (R Core Team, 2015). We used *asnipe* package (Farine, 2013) to construct a social network of wild great tit and blue tit populations. We first tested if the associations in the network were non-random by conducting permutation tests on the group matrix. The mean weighted degree of our network was significantly greater than values from the permutations (*p* = 0.001), demonstrating that our network differed significantly from random. We then calculated association between observers and demonstrators within our network of 331 individuals (217 blue tits and 114 great tits). Association strengths were scaled between 0 (never observed in the same flock) and 1 (always observed in the same flock), and in our experiment, these values ranged from 0 to 0.070 (mean = 0.018, sd = 0.021). In addition, we calculated the total number of interactions (i.e., times observed in the same flock) between demonstrators and observers: these ranged from 0 to 107 (mean = 24, sd = 30.803). As both association measures gave similar results when analysing observer’s behaviour, we decided to use only association strength in our final analyses.

We used a Wilcoxon signed ranked test to analyse differences in demonstrators’ behaviour when encountering aversive or palatable prey, to allow for the small sample size (*n* = 6). As the time that birds spent on foraging differed among demonstrators (palatable prey: range = 38–170 s, median = 77 s; aversive prey: range = 16–115 s, median = 62 s) we first divided the time spent on beak wiping and a number of beak wipes and head shakes with the total time foraging, and then compared these rates between aversive and palatable prey.

For analyses of observers’ behaviour, we next used generalized linear mixed effects models with appropriate error distributions, implemented using the *lme4* package (Bates et al., 2014). Explanatory variables in all models included an interaction between the effects of a demonstrator being present (cups before/demonstrator/cups after) with the prey type (palatable/aversive), the observer’s association with its demonstrator as determined from the social network, and the test order (first/second test). In addition, we included an observer’s identity and a demonstrator video as random effects. The baseline level of each model included an initial cup presentation, aversive prey type, and a first video presentation. As the length of time that birds were visible in videos differed, we modeled the time observers faced the screen or spent flying versus the length of time observers performed other behaviours (i.e., total time visible –time facing a video or flying) as a bound response variable with a binomial error distribution. Similarly, we converted the number of head turns and hops into a rate by dividing the number of times they occurred by the total time a bird was visible. We then converted these rates to integers by multiplying them by 30 s, which was the most common length of time a bird was visible during each section of the videos. We modeled head turns using a Poisson error distribution, but hopping with a negative binomial error distribution because it was right-skewed.

Finally, we analysed if social information in the videos affected which cup observers landed on first and how fast they made their choice, using again generalized linear mixed effects models. To test the effects of video on birds’ first choice, we included the choice (same/different cup that a demonstrator fed from) as a response variable, and prey type (palatable/aversive), the observer’s association with its demonstrator, and the test order as explanatory variables, using binomial error distribution. The baseline level of the model included the video playback of aversive prey and a first video presentation. Because the distribution of time before birds chose the cup was right-skewed, we modeled it with a negative binomial error distribution, using the time before a choice as a response variable, and an interaction between prey type (palatable/aversive), social information use (0/1, i.e., not matching/matching a demonstrator’s behaviour), and the test order as explanatory variables. The baseline level of the model included the video playback of aversive prey, a first video presentation and individuals that did not match a demonstrator’s behaviour. Bird identity and a demonstrator video were included as random effects in both models. Most of the birds landed on a cup during the first five minutes after cups were presented. Two individuals, however, were considerably slower at choosing in the aversive prey test, and landed on a cup only after 15 min. We therefore considered them as outliers, and removed them from the final analysis.

**Figure 2:**
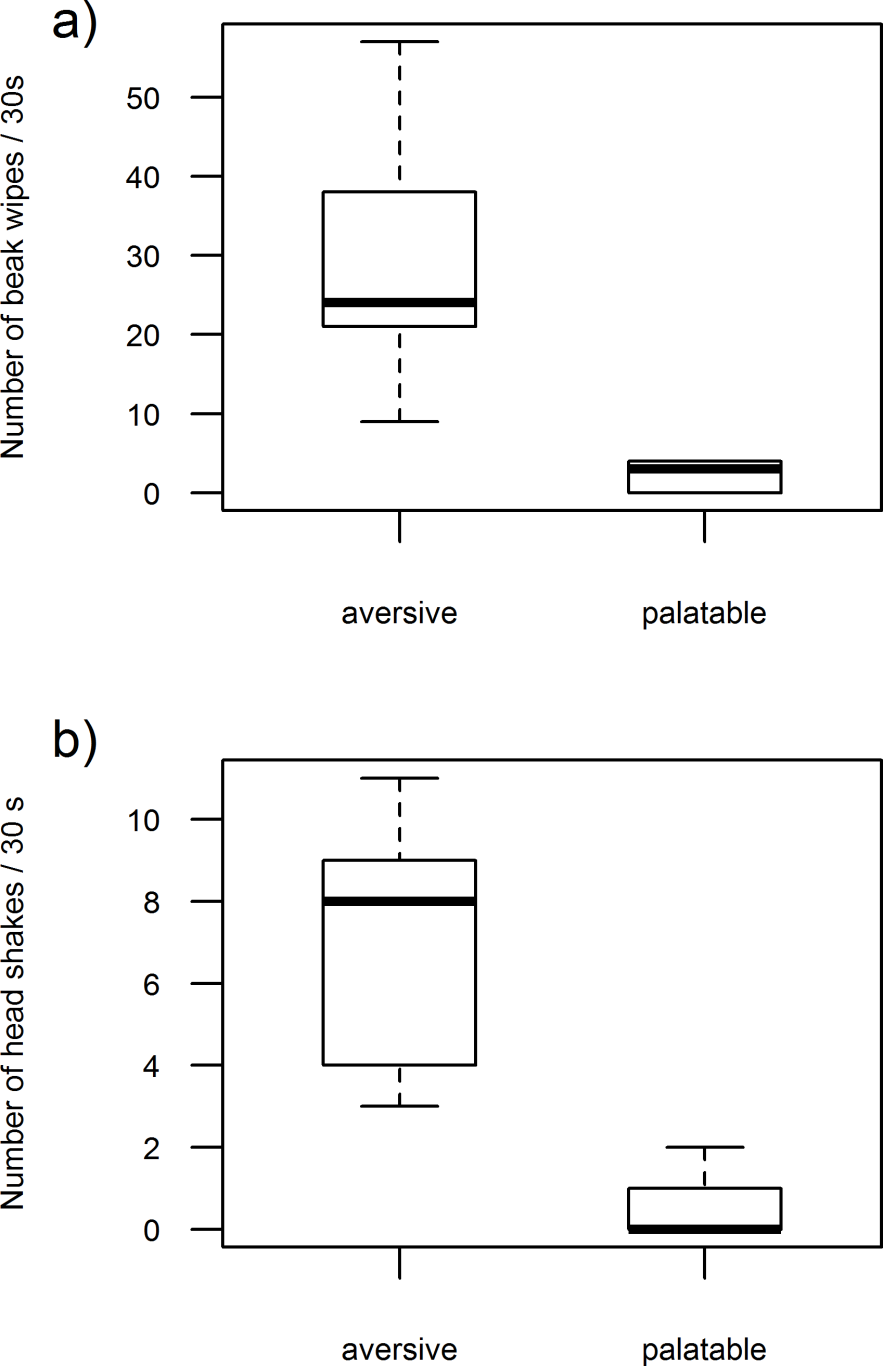
Demonstrators’ response. The rate of beak wipes (A) and headshakes (B) that demonstrators (*n* = 6) performed when encountering aversive or palatable prey.

## Results

### Demonstrators’ response

Demonstrators responded differently to palatable and aversive prey. The time demonstrators spent wiping their beaks on a perch (Wilcoxon signed-ranked test, *p* = 0.03) as well as the total number of beak wipes (Wilcoxon signed-ranked test, *p* = 0.03, Fig. 2A) were both significantly greater when demonstrators encountered aversive prey (time spent on beak wiping: median = 14 s, range = 4–23 s; number of beak wipes: median = 49, range = 20–107), compared to palatable prey (time spent on beak wiping: median = 1.5 s, range = 0–5 s; number of beak wipes: median = 5.5, range = 0–17). Demonstrators also performed more headshakes when eating aversive prey (median = 17.5, range = 5–27) than when eating palatable prey (Wilcoxon signed-ranked test, *p* = 0.03; Fig. 2B; median = 0, range = 0–5).

### Focal birds’ response to video-playback

#### Facing the screen

**Figure 3:**
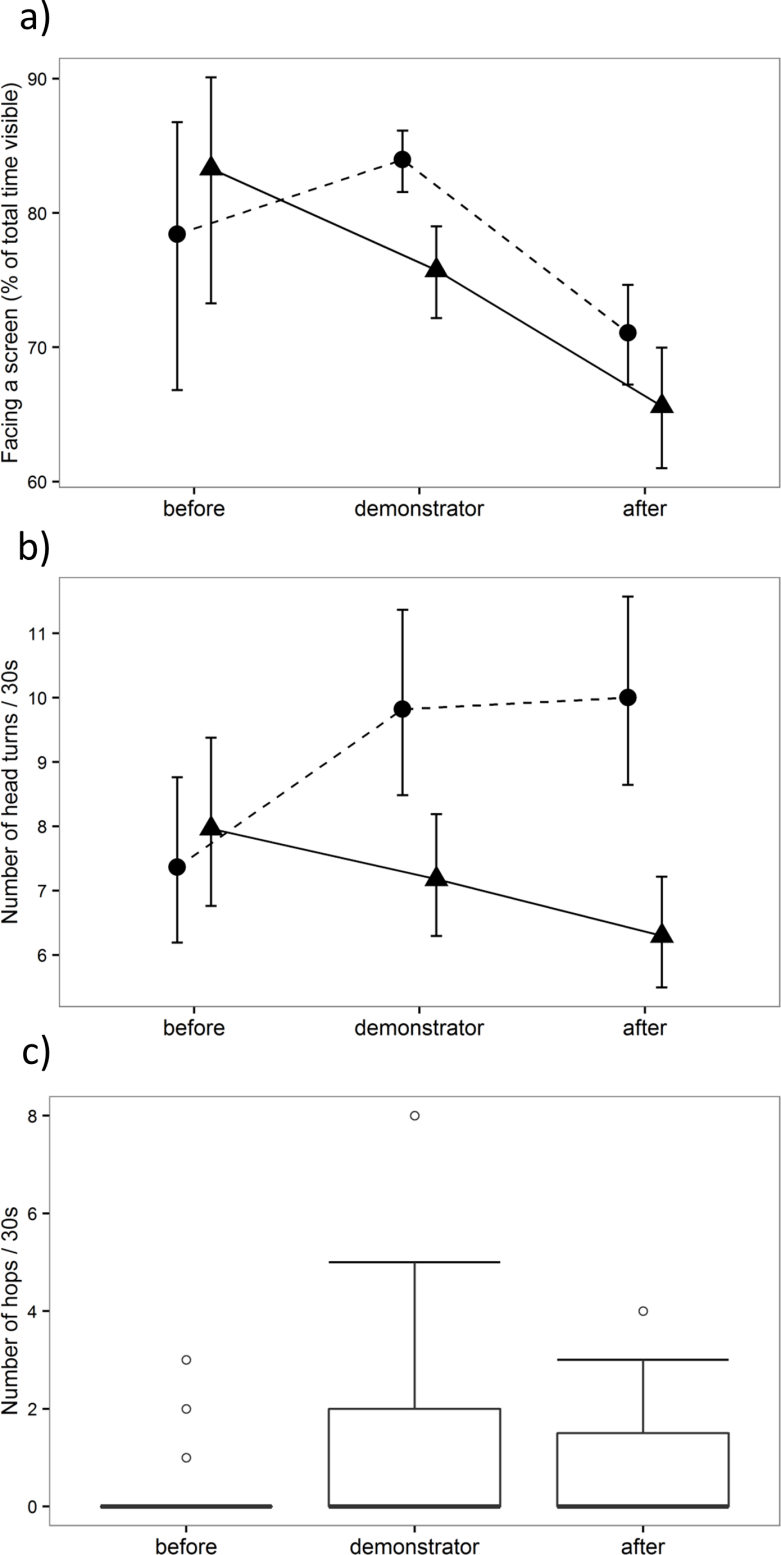
Focal birds’ response to video playback. Proportion of time observers faced the screen (A), and the rate of head turns (B) and hops (C) observers performed when they were presented (i) an empty cage with a feeding cup before a demonstrator, (ii) a demonstrator or (iii) an empty cage with a feeding cup after a demonstrator. The time observers faced the screen, and the number of head turns differed between palatable (triangles + solid line) and aversive prey (circles + dashed line). Graphs (A) and (B) show the means and standard errors. Graph (C) shows the median and 25th and 75th percentiles; the whiskers indicate the values within 1.5 times the interquartile range, and circles are outliers (included in the analyses).

The time observers faced the video screen depended on the prey type a demonstrator encountered (Fig. 3A): observers faced the screen less during (demonstrator presence * prey type; estimate = − 0.836 ± 0.252, *Z* =  − 3.320, *p* < 0.001) and after (cups after demonstrator playback * prey type: estimate = − 0.570 ± 0.269, *Z* =  − 2.118, *p* = 0.03) the presentation of palatable prey compared to aversive prey. There were no significant differences between video types in the way observers responded to cups only before the presentation of a demonstrator (estimate =0.318 ± 0.720, *Z* = 0.441, *p* = 0.66), suggesting that it was the behaviour of the demonstrator that influenced how long observers faced the video screen. Following presentation of any demonstrators, observers paid overall less attention to the screen showing cups only (estimate = − 0.392 ± 0.181, *Z* =  − 2.167, *p* = 0.03; Fig. 3A). Focal birds also faced the screen more during the second test (estimate =0.386 ± 0.144, *Z* = 2.679, *p* = 0.007). In addition, association strength with a demonstrator had a significant effect on focal birds’ behaviour, showing that individuals faced the screen less when they were more closely associated with a demonstrator (estimate = − 28.099 ± 12.296, *Z* =  − 2.285, *p* = 0.022). The distribution of association scores, however, was skewed, as most of the individuals had low association scores with a demonstrator, and it is therefore difficult to interpret this result. Finally, bird identity (variance = 0.674) and demonstrator video (variance = 1.417), included to the model as random effects, explained some of the observed variation. The final model is presented in File S1, Table 1.

#### Flying

Although observers varied in how much time they spent flying during video playback (range =0–29 s, mean = 4.8 s), there was no effect of demonstrator presence and prey type on this behaviour (compared to initial cup presentation, demonstrator present * prey type: estimate =0.185 ± 0.289, *Z* = 0.640, *p* = 0.52; cups after demonstrator playback * prey type: estimate =0.169 ± 0.310, *Z* = 0.544, *p* = 0.59) and their interactions were therefore removed from the final model. The final model showed that a demonstrator’s presence, regardless of prey type (estimate = − 0.248 ± 0.144, *Z* =  − 1.729, *p* = 0.16), the test order (estimate =0.114 ± 0.158, *Z* = 0.718, *p* = 0.47) or the association between observer and demonstrator (estimate =14.913 ± 13.957, *Z* = 1.069, *p* = 0.29) did not affect the time that observers were flying during video playback, but random effects explained some of the observed variation (variance for bird identity =0.908; variance for demonstrator video =0.987; Table 2 in File S1).

### Head turns

The number of head turns observers performed depended on a demonstrator’s presence in the video and prey type a demonstrator encountered (Fig. 3B). Observers performed fewer head turns during (demonstrator presence * prey type; estimate = − 0.393 ± 0.196, *Z* =  − 2.002, *p* = 0.045) and after (cups after demonstrator playback * prey type: estimate = − 0.543 ± 0.199, *Z* =  − 2.726, *p* = 0.006) the presentation of palatable prey compared to aversive prey. During the initial cup presentation, observers’ responses did not differ significantly between these video types (estimate =0.015 ± 0.278, *Z* = 0.055, *p* = 0.96). The test order (estimate =0.188 ± 0.141, *Z* = 1.337, *p* = 0.181) and association between demonstrator and observer (estimate = − 8.142 ± 5.062, *Z* =  − 1.608, *p* = 0.11) had no effect on the number of head turns performed. In addition, the variance estimates for the random effects were small (variance for bird identity =0.081; variance for demonstrator video =0.158; Table 3 in File S1).

#### Hops

The number of hops that observers performed did not depend on the prey type a demonstrator encountered (compared to initial cup presentation, demonstrator present * prey type: estimate = − 1.375 ± 0.951, *Z* =  − 1.446, *p* = 0.15; cups after demonstrator playback * prey type: estimate = − 0.636 ± 1.015, *Z* =  − 0.626, *p* = 0.53), so we removed these interactions from the final model. The final model shows that birds were hopping significantly more in the presence of a demonstrator compared to initial cup presentation (estimate =1.967 ± 0.565, *Z* = 3.482, *p* < 0.001; Fig. 3C) and to the cup presentation after a demonstrator (estimate =0.953 ± 0.460, *Z* = 2.071, *p* = 0.04). Again, the test order (estimate =0.135 ± 0.452, *Z* = 0.297, *p* = 0.77) and the association with a demonstrator (estimate =6.237 ± 10.131, *Z* = 0.616, *p* = 0.54) had no effect on an observer’s behaviour, and the variance estimates for random effects were small (variance for bird identity <  0.001; variance for demonstrator video <  0.001; Table 4 in File S1). One individual hopped considerably more than the others, but re-running analyses without it did not change the results. In particular, the increase in hopping in the presence of a demonstrator remained significant (hops during presence of a demonstrator versus initial cup presentation: estimate =1.653 ± 0.415, *Z* = 3.981, *p* < 0.001).

### Social information use

Prey type in the video (estimate =0.372 ± 0.814, *Z* = 0.457, *p* = 0.65), the test order (estimate =0.829 ± 0.745*Z* = 1.113, *p* = 0.27) and the association score with a demonstrator (estimate = − 17.137 ± 14.739, *Z* =  − 1.163, *p* = 0.25) did not have a significant effect on an observer’s cup choice, but demonstrator video, included as a random effect, explained some of the observed variation (variance for bird identity =0.031; variance for demonstrator video =0.442; Table 5 in File S1). After observing a demonstrator discover palatable prey in a coloured cup, only 10 birds landed first on that cup, whereas 10 birds chose the alternative coloured cup (binomial test, 10/20 compared to equal probability, *p* = 1). Similarly, after video-playback of a demonstrator’s response towards aversive prey, only 12 birds avoided the cup that the demonstrator fed from, whereas nine birds landed on it first (binomial test, 9/21 compared to equal probability, *p* = 0.66). Only five birds matched our predictions in both tests, choosing the same cup colour as a demonstrator after receiving information about palatable prey, and avoiding that colour after seeing a demonstrator’s disgust response. Again, this was not different from what would be expected if birds foraged randomly (binomial test, 5/20 compared to probability of 0.25, *p* = 1).

**Figure 4:**
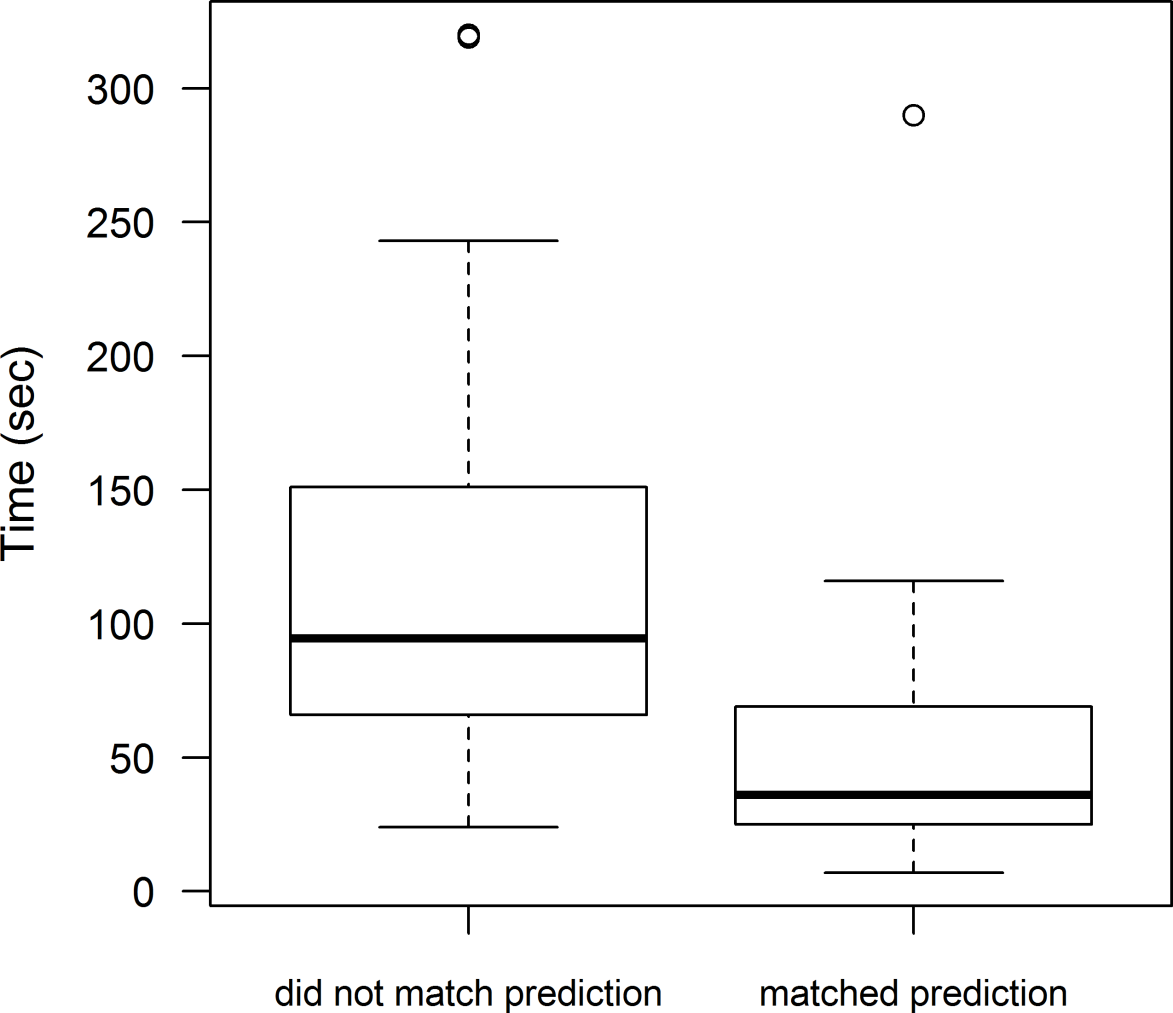
Time before individuals chose a cup in a two-choice test. Time (s) before individuals made their cup choice when they (i) chose a different cup than predicted (i.e., did not use social information, *n* = 19), or (ii) chose the predicted cup (*n* = 21). The interaction between prey type and social information use did not have a significant effect on time that it took birds to choose a cup (estimate =0.218 ± 0.532, *Z* = 0.410, *p* = 0.68), so responses are plotted across prey types. The box plots show the median and 25th and 75th percentiles; the whiskers indicate the values within 1.5 times the interquartile range, and circles are outliers (included in the analyses).

Information in the video, however, did appear to affect the latency of observers’ cup choice. Observers made their choice faster when they chose a cup matching the social information provided in the video (compared to birds that did not match our predictions, estimate = − 0.837 ± 0.265, *Z* =  − 3.154, *p* = 0.002; Fig. 4). This did not vary between the prey types observed (estimate = − 0.024 ± 0.262, *Z* =  − 0.092, *p* = 0.93), or between the first and the second test (estimate =0.009 ± 0.226, *Z* = 0.043, *p* = 0.97). Therefore, in both tests birds chose the cup faster when their decision matched a demonstrator’s behaviour. The variance estimates for random effects were small (variance for bird identity =0.167; variance for demonstrator video =0.062; Table 6 in File S1).

## Discussion

Our experiment shows that blue tits, like many other species tested (e.g., McQuoid & Galef, 1993; Rieucau & Giraldeau, 2009; Zoratto et al., 2014) pay attention to video playback of a conspecific. Focal birds were more active in the presence of a demonstrator than when videos showed a cage without a conspecific present, and observers’ behaviour differed depending on which prey type was being sampled in the demonstration video. Furthermore, the identity of the demonstrator also explained some variation in observers’ behaviour. Despite our predictions, however, we found that only half of the focal birds then chose to forage from the cup according to the information that demonstrators had provided, the same as if birds foraged randomly. Information from video playback, however, did influence how quickly birds made a choice: those that chose in accordance with the demonstrator did so more quickly. Together, these results suggest that social cues in video playback could provide blue tits with information about prey profitability, but the birds either did not acquire or chose not to use this social information in their decision-making.

Similar to previous studies, (Clark, 1970; Skelhorn, 2011), we found that demonstrators responded to aversive prey by performing beak wipes and headshakes. Head shaking and beak wiping has been proposed to provide observers with information about potentially toxic prey (Johnston, Burne & Rose, 1998; Skelhorn, 2011). This kind of ‘disgust’ behaviour could increase the benefits for paying attention to a demonstrator, as encounters with aversive prey can elevate internal toxin levels (Skelhorn, Halpin & Rowe, 2016) or even increase the risk of mortality when prey are lethal (Longson & Joss, 2006). Perhaps this explains why we observed differences in focal birds’ behaviour between different video types, and why observers continued to pay more attention to a cup in an empty cage after observing a demonstrator encounter aversive prey. Alternatively, it is possible that focal birds faced the video screen and made more head turns when demonstrators discovered aversive prey simply because demonstrators were more active during this presentation. This could be investigated further by presenting focal birds videos that contain different cues but show similar amounts of activity.

Other research on social information use by blue tits also finds that use of social information is low: only approximately 50% of individuals learn to solve a novel feeding task after observing a conspecific solve it (Sasvári, 1979; Aplin, Sheldon & Morand-Ferron, 2013). These studies differ from our experiment because in task solving tests, none of the individuals are expected to solve the task without demonstration or training; in our experiment, individuals had a 50% probability to choose the predicted cup just by chance. Our result, that focal birds’ foraging decisions do not differ from what would be expected if birds were choosing the cup randomly, is difficult to interpret. Individuals that chose the predicted cup in our experiment may have used social information, or may have just chosen by chance a cup that matched a demonstrator’s behaviour. However, we found that individuals made their choice faster when choosing a cup that matched the information in the video. It has been suggested that one of the costs of personal information use is time and energy that individuals need for information gathering (Dall et al., 2005), and the observed time difference in our experiment indicates that the time cost to make a decision might be higher if individuals do not use social information. However, if we assume that all birds that chose the cup according to our predictions used social information because they made their choices more quickly, it is difficult to explain why all the other birds would have chosen an alternative cup, as we would expect their choices to be random. Alternatively, if we assume that none of the birds used social information from the videos, it is difficult to explain the observed difference in their decision times.

One possibility is that some of the focal birds used the cues from videos differently than others. For example, neophobia or competition could have affected observers’ foraging choices (Gibelli & Dubois, 2017). After seeing a demonstrator eating palatable prey from a coloured cup, some observers might interpret that cup to be empty and therefore choose an alternative cup to avoid competition. On the other hand, observers might interpret the video of a cup in an empty cage so that demonstrators did not want to forage from that cup. Individuals might therefore choose the same cup as a demonstrator just to avoid novelty, even after seeing a demonstrator eating aversive prey. Furthermore, birds were pre-trained to locate favourable prey items from food cups, albeit of different colour, so instead of using social information about food unpalatability, they might have relied on their previous experience with prey (e.g., Kendal, Coolen & Laland, 2004). In addition, individuals might have had preferences for different cup colours, but we did not find that they chose any of the coloured cups more often than others, and the cup colours for demonstrator and control videos were randomized.

It has been suggested that individuals are more likely to rely on social information when the costs to acquire personal information increase (Kendal, Coolen & Laland, 2004; Kendal et al., 2005), and it is possible that in our experiment the cost of foraging from the “wrong” cup was too low to detect information use. In addition, some characteristics of observers such as sex and age (Loukola, Seppänen & Forsman, 2012; Aplin, Sheldon & Morand-Ferron, 2013; Guillette & Healy, 2014), personality (Marchetti & Drent, 2000), or dominance status (Nicol & Pope, 1999), could have made it more difficult for us to detect an effect of social information on cup choice. In a previous study of social learning in blue tits, for example, juvenile females were almost twice as likely to learn the novel foraging task as other age/sex classes, and only 37.5% of adults overall learned by observing others (Aplin, Sheldon & Morand-Ferron, 2013). In our study, all observers were adult birds and we do not know their sex. Future work should therefore present blue tits with more complicated tests with higher foraging costs (e.g., Aplin, Sheldon & Morand-Ferron, 2013), and ensure sex and age are known. Still, in our experiment only five birds made their choice in accordance with the demonstrator in both tests, so individuals were not consistent in their information use and it is therefore unlikely that their sex would explain the observed variation. The variation in information use between the two tests, however, is not surprising, as individuals might value different types of social information differently. For instance, individuals’ current toxin levels and energetic state might influence their decision to attack aversive prey (Skelhorn, Halpin & Rowe, 2016), and also increase the value of social information about prey unpalatability.

To further investigate the effectiveness of video playback in parid tits, it could be useful to compare focal birds’ responses to videos to their response to live demonstrators. This, however, would be difficult to conduct as live demonstrators vary in when and how they perform behaviours. For example, individuals’ propensity to eat aversive prey could depend on intrinsic differences, such as their current energetic state (Skelhorn, Halpin & Rowe, 2016) that might vary over time. In our experiment, the time that demonstrators spent eating aversive prey differed considerably among individuals, and some of the demonstrators showed a stronger disgust response (more beak wipes and head shakes) than others. With video playback, we could present focal birds standardized videos of demonstrators’ responses but the use of live demonstrators would include much more variation, and therefore require a high number of individuals to be tested. A second potential problem with our experiment was that vocal communication between observer and demonstrator was impossible. However, we did not hear any of the focal birds vocalize during playback, nor did any of the demonstrators vocalize during filming. We therefore suggest that our results of focal birds’ behavioural changes during video playback, the different responses depending on demonstrator identity, and the effect we detected on the latency to forage, provide adequate evidence that blue tits pay attention to video playback. We assert that this could be a valid method for studying social information use.

### Conclusion

In conclusion, our study showed that blue tits respond to video playback of a conspecific, and that individuals paid more attention to demonstrators encountering aversive prey. This indicates that they did not only respond to the presence of a demonstrator but also observed differences in a demonstrator’s behaviour. The cues from videos then influenced focal birds’ behaviour in a foraging task, as individuals that chose to forage in accordance with a demonstrator made their foraging choice faster. The proportion of birds that made their choice according to information from videos, however, did not differ from random, and we are therefore unable to explain the differences in these two measures of foraging. Together, our results suggest that video playback of a conspecific can provide social cues to blue tits, and video playback therefore provides a promising method for studying social behaviour and learning in parid tits, with potential application for studies in both captivity and the wild. However, we do not know how these social cues are later used in decision-making, and this seems to vary greatly among individuals.

##  Supplemental Information

10.7717/peerj.3062/supp-1 Video S1 Demonstrator encountering aversive prey The video shows a demonstrator encountering aversive prey (mealworm injected with bitter-tasting Bitrex).

10.7717/peerj.3062/supp-2 Video S2 Demonstrator encountering palatable prey The video shows a demonstrator encountering palatable prey (mealworm).

10.7717/peerj.3062/supp-3 Video S3 Observer’s response to video playback The video shows an observer’s response to different sections of video playback: (i) a control section (a cup in an empty cage) before a demonstrator (30 s), (ii) a demonstrator encountering aversive prey (45 s), and (iii) a control section (a cup in an empty cage) after a demonstrator (30 s).

10.7717/peerj.3062/supp-4 File S1 Results of the GLM models More comprehensive presentation of the results from all GLM models described in the results section.

10.7717/peerj.3062/supp-5 Data S1 Raw data used in the analyses

## Additional Information and Declarations

### Data Availability

The following information was supplied regarding data availability: The raw data has been supplied as Supplementary File.

## References

[ref-1] Aplin LM, Sheldon BC, Morand-Ferron J (2013). Milk bottles revisited: social learning and individual variation in the blue tit, *Cyanistes caeruleus*. Animal Behaviour.

[ref-2] Bates D, Mächler M, Bolker B, Walker S (2014). Fitting linear mixed-effects models using lme4. Journal of Statistical Software.

[ref-3] Bird CD, Emery NJ (2008). Using video playback to investigate the social preferences of rooks, *Corvus frugilegus*. Animal Behaviour.

[ref-4] Burford FRL, McGregor PK, Oliveira RF (2000). Response of fiddler crabs (*Uca tangeri*) to video playback in the field. Acta Ethologica.

[ref-5] Carter AJ, Marshall HH, Heinsohn R, Cowlishaw G (2014). Personality predicts the propensity for social learning in a wild primate. PeerJ.

[ref-6] Chandrashekar J, Mueller KL, Hoon MA, Adler E, Feng L, Guo W, Zuker CS, Ryba NJ (2000). T2Rs function as bitter taste receptors. Cell.

[ref-7] Clark GJ (1970). Avian bill-wiping. The Wilson Bulletin.

[ref-8] Clark DL, Macedonia JM, Rosenthal GG (1997). Testing video playback to lizards in the field. Copeia.

[ref-9] Clark DL, Uetz GW (1992). Morph-independent mate selection in adimorphic jumping spider: demonstration of movement bias in female choice using video-controlled courtship behaviour. Animal Behaviour.

[ref-10] Dall SR, Giraldeau LA, Olsson O, McNamara JM, Stephens DW (2005). Information and its use by animals in evolutionary ecology. Trends in Ecology & Evolution.

[ref-11] D’Eath RB (1998). Can video images imitate real stimuli in animal behaviour experiments?. Biological Reviews.

[ref-12] Ekman J (1989). Ecology of non-breeding social systems of *Parus*. The Wilson Bulletin.

[ref-13] Evans CS, Macedonia JM, Marler P (1993). Effects of apparent size and speed on the response of chickens, *Gallus gallus*, to computer-generated simulations of aerial predators. Animal Behaviour.

[ref-14] Farine DR (2013). Animal social network inference and permutations for ecologists in R using asnipe. Methods in Ecology and Evolution.

[ref-15] Farine DR, Spencer KA, Boogert NJ (2015). Early-life stress triggers juvenile zebra finches to switch social learning strategies. Current Biology.

[ref-16] Firth JA, Sheldon BC, Farine DR (2016). Pathways of information transmission among wild songbirds follow experimentally imposed changes in social foraging structure. Biology Letters.

[ref-17] Franks DW, Ruxton GD, James R (2010). Sampling animal association networks with the gambit of the group. Behavioral Ecology and Sociobiology.

[ref-18] Fryday SL, Greig-Smith PW (1994). The effects of social learning on the food choice of the house sparrow (*Passer domesticus*). Behaviour.

[ref-19] Galef BG, Giraldeau LA (2001). Social influences on foraging in vertebrates: causal mechanisms and adaptive functions. Animal Behaviour.

[ref-20] Ganchrow JR, Steiner JE, Bartana A (1990). Behavioral reactions to gustatory stimuli in young chicks (*Gallus gallus domesticus*). Developmental Psychobiology.

[ref-21] Gibelli J, Dubois F (2017). Does personality affect the ability of individuals to track and respond to changing conditions?. Behavioral Ecology.

[ref-22] Guillette LM, Healy SD (2014). Mechanisms of copying behaviour in zebra finches. Behavioural Processes.

[ref-23] Gunhold T, Whiten A, Bugnyar T (2014). Video demonstrationsseed alternative problem-solving techniques in wild common marmosets. Biology Letters.

[ref-24] Heinrich B (1988). Why do ravens fear their food?. The Condor.

[ref-25] Johnston A, Burne T, Rose S (1998). Observation learning in day-old chicks using a one trial passive avoidance learning paradigm. Animal Behaviour.

[ref-26] Kendal RL, Coolen I, Laland KN (2004). The role of conformity in foraging when personal and social information conflict. Behavioral Ecology.

[ref-27] Kendal RL, Coolen I, Van Bergen Y, Laland KN (2005). Trade-offs in the adaptive use of social and asocial learning. Advances in the Study of Behavior.

[ref-28] Longson CG, Joss JMP (2006). Optimal toxicity in animals: predicting the optimal level of chemical defences. Functional Ecology.

[ref-29] Loukola OJ, Seppänen JT, Forsman JT (2012). Intraspecific social information use in the selection of nest site characteristics. Animal Behaviour.

[ref-30] Marchetti C, Drent PJ (2000). Individual differences in the use of social information in foraging by captive great tits. Animal Behaviour.

[ref-31] Mason JR, Reidinger RF (1981). Effects of social facilitation and observational learning on feeding behavior of the red-winged blackbird (*Agelaius phoeniceus*). The Auk.

[ref-32] Mason JR, Reidinger RF (1982). Observational learning of food aversions in red-winged blackbirds (*Agelaius phoeniceus*). The Auk.

[ref-33] McQuoid LM, Galef BG (1993). Social stimuli influencing feeding behaviour of Burmese fowl: video analysis. Animal Behaviour.

[ref-34] Mesoudi A, Chang L, Dall SR, Thornton A (2016). The evolution of individual and cultural variation in social learning. Trends in Ecology & Evolution.

[ref-35] Nicol CJ, Pope SJ (1999). The effects of demonstrator social status and prior foraging success on social learning in laying hens. Animal Behaviour.

[ref-36] Ophir AG, Galef BG (2003). Female Japanese quail affiliate with live males that they have seen mate on video. Animal Behaviour.

[ref-37] Ord TJ, Peters RA, Evans CS, Taylor AJ (2002). Digital video playback and visual communication in lizards. Animal Behaviour.

[ref-38] Psorakis I, Roberts SJ, Rezek I, Sheldon BC (2012). Inferring social network structure in ecological systems from spatio-temporal data streams. Journal of the Royal Society Interface.

[ref-39] Pyke GH, Pulliam HR, Charnov EL (1977). Optimal foraging: a selective review of theory and tests. Quarterly Review of Biology.

[ref-40] R Core Team A (2015). R: a language and environment for statistical computing.

[ref-41] Rieucau G, Giraldeau LA (2009). Video playback and social foraging: simulated companions produce the group size effect in nutmeg mannikins. Animal Behaviour.

[ref-42] Rowland WJ, Bolyard KJ, Jenkins JJ, Fowler J (1995). Video playback experiments on stickleback mate choice: female motivation and attentiveness to male colour cues. Animal Behaviour.

[ref-43] Sasvári L (1979). Observational learning in great, blue and marsh tits. Animal Behaviour.

[ref-44] Skelhorn J (2011). Colour biases are a question of conspecifics’taste. Animal Behaviour.

[ref-45] Skelhorn J, Halpin CG, Rowe C (2016). Learning about aposematic prey. Behavioral Ecology.

[ref-46] Skelhorn J, Rowe C (2009). Distastefulness as an antipredator defence strategy. Animal Behaviour.

[ref-47] Woo KL, Rieucau G From dummies to animations: a review of computer-animated stimuli used in animal behavior studies. Behavioral Ecology and Sociobiology.

[ref-48] Zoratto F, Manzari L, Oddi L, Pinxten R, Eens M, Santucci D, Alleva E, Carere C (2014). Behavioural response of European starlings exposed to video playback of conspecific flocks: effect of social context and predator threat. Behavioural Processes.

