# Supplementary material for: Can video playback provide social information for foraging blue tits?
Source: PeerJ. 2017 Mar 21;5:e3062. doi: 10.7717/peerj.3062 (PMC5363260; doi:10.7717/peerj.3062)
Supplement: File S1 — More comprehensive presentation of the results from all GLM models described in the results section. [file peerj-05-3062-s004.docx]

**SUPPLEMENTARY MATERIAL FOR**

**Can video playback provide social information for foraging blue tits?**

Liisa Hämäläinen, Hannah M. Rowland, Johanna Mappes and Rose Thorogood

**Results of the GLM models**

**Table 1.** GLMM explaining the time focal birds faced a screen during video playback. Intercept gives the estimate for the time birds faced a screen when they were shown a cup before a demonstrator in the first test, and video playback of aversive prey. Bird ID (variance = 0.674) and demonstrator video (variance = 1.417) were included as random effects.

|  |  |  |  |  |  |  |  |  |  |
| --- | --- | --- | --- | --- | --- | --- | --- | --- | --- |
| **Source** |  |  | **Estimate** |  | **SE** |  | **Z** |  | **P** |
| Intercept |  |  | 1.2901 |  | 0.5902 |  | 2.186 |  | 0.0288 |
| Cup after |  |  | - 0.3916 |  | 0.1807 |  | -2.167 |  | 0.0302 |
| Demonstrator | |  | 0.3664 |  | 0.1694 |  | 2.162 |  | 0.0306 |
| Palatable prey | |  | 0.3175 |  | 0.7203 |  | 0.441 |  | 0.6594 |
| Second test | |  | 0.3856 |  | 0.1440 |  | 2.679 |  | 0.0074 |
| Association score | |  | -28.0998 |  | 12. 2961 |  | -2.285 |  | 0.0223 |
| Cup after: palatable prey | | | -0.5696 |  | 0.2689 |  | -2.118 |  | 0.0342 |
| Demonstrator: palatable prey | | | -0.8356 |  | 0.2517 |  | -3.320 |  | <0.001 |
|  | | |  |  |  |  |  |  |  |

**Table 2.** GLMM explaining the time focal birds were flying in a cage during video playback. Intercept gives the estimate for the time birds were flying when they were shown a cup before a demonstrator in a first test. Bird ID (variance = 0.9081) and demonstrator video (variance = 0.9873) were included as random effects.

|  |  |  |  |  |  |  |  |  |  |
| --- | --- | --- | --- | --- | --- | --- | --- | --- | --- |
| **Source** |  |  | **Estimate** |  | **SE** |  | **Z** |  | **P** |
| Intercept |  |  | -2.3551 |  | 0.4794 |  | -4.913 |  | < 0.0001 |
| Cup after |  |  | -0.0318 |  | 0.1537 |  | -0.207 |  | 0.836 |
| Demonstrator | |  | -0.2484 |  | 0.1436 |  | -1.729 |  | 0.157 |
| Second test | |  | 0.1135 |  | 0.1580 |  | 0.718 |  | 0.473 |
| Association score | |  | 14. 9130 |  | 13.9566 |  | 1.069 |  | 0.2853 |
|  | |  |  |  |  |  |  |  |  |

**Table 3.** GLMM explaining the number of head turns focal birds performed during video playback. Intercept gives the estimate for the number of head turns when birds were shown a cup before a demonstrator in a first test, and video playback of aversive prey. Bird ID (variance = 0.0812) and demonstrator video (variance = 0.1577) were included as random effects.

|  |  |  |  |  |  |  |  |  |  |
| --- | --- | --- | --- | --- | --- | --- | --- | --- | --- |
| **Source** |  |  | **Estimate** |  | **SE** |  | **Z** |  | **P** |
| Intercept |  |  | 1.9046 |  | 0.2325 |  | 8.192 |  | < 0.0001 |
| Cup after |  |  | 0.3060 |  | 0.1455 |  | 2.103 |  | 0.0355 |
| Demonstrator | |  | 0.2877 |  | 0.1461 |  | 1.969 |  | 0.0489 |
| Palatable prey | |  | 0.0153 |  | 0.2776 |  | 0.055 |  | 0.9561 |
| Second test | |  | 0.1880 |  | 0.1406 |  | 1.337 |  | 0.1811 |
| Association score | |  | -8.1419 |  | 5.0620 |  | -1.608 |  | 0.1077 |
| Cup after x positive video | | | -0.5427 |  | 0.1991 |  | -2.726 |  | 0.0064 |
| Demonstrator x positive video | | | -0.3933 |  | 0.1964 |  | -2.002 |  | 0.0452 |
|  | | |  |  |  |  |  |  |  |

**Table 4.** GLMM explaining the number of hops focal birds performed during video playback. Intercept gives the estimate for the number of hops when birds were shown a cup before a demonstrator in a first test. Bird ID (variance < 0.001) and demonstrator video (variance < 0.001) were included as random effects.

| **Source** |  |  | **Estimate** |  | **SE** |  | **Z** |  | **P** |
| --- | --- | --- | --- | --- | --- | --- | --- | --- | --- |
| Intercept |  |  | -1.8701 |  | 0.6185 |  | -3.023 |  | 0.0025 |
| Cup after |  |  | 1.0146 |  | 0.5859 |  | 1.732 |  | 0.0833 |
| Demonstrator | |  | 1.9673 |  | 0.5650 |  | 3.482 |  | 0.0005 |
| Second test | |  | 0.1345 |  | 0.4524 |  | 0.297 |  | 0.7662 |
| Association score | |  | 6.2373 |  | 10.1306 |  | 0.616 |  | 0.5381 |
|  | |  |  |  |  |  |  |  |  |

**Table 5.** GLMM explaining focal birds’ first cup choice (same/different cup that a demonstrator fed from) after video playback. Explanatory variables include information type and test order (baseline level includes video of aversive prey and first test). Bird ID (variance = 0.0310) and demonstrator video (variance = 0.4419) were included as random effects.

| **Source** |  |  | **Estimate** |  | **SE** |  | **Z** |  | **P** |
| --- | --- | --- | --- | --- | --- | --- | --- | --- | --- |
| Intercept |  |  | -0.3426 |  | 0.6968 |  | -0.492 |  | 0.623 |
| Palatable prey video |  |  | 0.3719 |  | 0.8135 |  | 0.457 |  | 0.648 |
| Second test | |  | 0.8294 |  | 0.7454 |  | 1.113 |  | 0.266 |
| Association score | |  | -17.1373 |  | 14.7398 |  | -1.163 |  | 0.245 |
|  | |  |  |  |  |  |  |  |  |

**Table 6.** GLMM explaining the latency to choose a cup after video playback. Intercept gives the estimate for the time (in seconds) that it took for focal birds to choose a cup when their choice did not match a demonstrator’s behaviour, and when they saw video playback of aversive prey in the first test. Bird ID (variance = 0.1668) and demonstrator video (variance = 0.0616) were included as random effects.

| **Source** |  |  | **Estimate** |  | **SE** |  | **Z** |  | **P** |
| --- | --- | --- | --- | --- | --- | --- | --- | --- | --- |
| Intercept |  |  | 4.6607 |  | 0.2729 |  | 17.075 |  | < 0.001 |
| Palatable prey video |  |  | -0.0241 |  | 0.2620 |  | -0.092 |  | 0.9266 |
| Second test | |  | 0.0098 |  | 0.2258 |  | 0.043 |  | 0.9655 |
| Matching demonstrator | |  | -0.8368 |  | 0.2653 |  | -3.154 |  | 0.0016 |
|  | |  |  |  |  |  |  |  |  |
